# Durability of pulmonary vein isolation following cryoballoon ablation: Lessons from a large series of repeat ablation procedures

**DOI:** 10.1016/j.ijcha.2022.101040

**Published:** 2022-04-27

**Authors:** Giacomo Mugnai, Federico Cecchini, Erwin Stroker, Gaetano Paparella, Saverio Iacopino, Juan Sieira, Yves De Greef, Luca Tomasi, Bruna Bolzan, Gezim Bala, Ingrid Overeinder, Alexandre Almorad, Anais Gauthey, Antonio Sorgente, Flavio Luciano Ribichini, Carlo de Asmundis, Gian-Battista Chierchia

**Affiliations:** a Heart Rhythm Management Centre, Universitair Ziekenhuis Brussel, Postgraduate Program in Cardiac Electrophysiology and Pacing, European Reference Networks Guard-Heart, Vrije Universiteit Brussel, Brussels, Belgium; b Electrophysiology and Cardiac Pacing, Division of Cardiology, Department of Medicine, University Hospital of Verona, Verona, Italy; c Electrophysiology Unit, ZNA Middelheim, Antwerp, Belgium

**Keywords:** Cryoballoon, Pulmonary vein isolation, Reconnections, Conduction gaps

## Abstract

**Introduction:**

The second-generation cryoballoon (CB) has emerged in the last decade as an effective treatment for atrial fibrillation (AF). This study sought to analyze the rate of PV reconnection following CB ablation, evaluate the most frequent PV sites of conduction recovery and finally to assess procedural and biophysical indicators of reconnection in a large cohort of patients undergoing repeat ablation for recurrence of atrial arrhythmias.

**Methods and Results:**

A total of 300 consecutive patients (189 males, 63%; mean age 63.0 ± 11.1 years) underwent a repeat ablation after 18.2 ± 10.8 months from the index CB ablation. All repeat ablations were performed using a 3-dimensional electro-anatomical mapping system. Among all 1178 PVs, 209 (17.7%) showed a late PV reconnection in 177 patients (1.18 per patient), at the time of repeat ablation procedure. Overall, persistent PV isolation could be documented in 969 of 1178 PVs (82.3%). In 123 of 300 patients (41%), persistent isolation could be demonstrated in all PVs, whereas PV reconnection could be documented in 177 patients (59%). In the multivariable analysis, nadir temperature (p = 0.03), time to PV isolation (p = 0.01) and failure to achieve − 40 °C within 60 s (p = 0.05) were independently associated with late PV reconnection.

**Conclusions:**

The rate of late PV reconnection after CB ablation was low (1.18 PVs/patient). The most frequent sites of reconnections were the superior-anterior portions for the upper PVs and the inferior-posterior portions for the lower PVs. Faster time to isolation, colder nadir temperatures and achievement of − 40 °C within 60 s were associated with durable PV isolation.

## 1 Introduction

Pulmonary vein (PV) isolation is nowadays an established treatment for drug-resistant atrial fibrillation (AF) [1]. In the last decade, cryoballoon (CB) ablation emerged as a valid alternative to radiofrequency (RF) ablation [2]. The former is associated with more reproducible procedural times and might be less influenced by operator skills if compared to the traditional point-by-point approach [3]. The second-generation CB (Arctic Front Advance, Medtronic, MN) has been shown to achieve similar results compared with RF ablation in terms of clinical outcome and procedural safety [4], [5], [6], [7]. Animal studies have also confirmed the safety and efficacy of the second-generation CB [8], [9]. Despite encouraging results, arrhythmic recurrences after the index procedure remain relatively frequent which, in most of cases, might be related to PV reconnections, potentially reflecting the lack of efficacy in achieving transmurality and homogeneous long-lasting lesions.

To the best of our knowledge, little and controversial information is still available about the occurrence of late PV reconnection after CB ablation [10], [11], [12], [13], [14], [15], [16], [17]. Therefore, the present study addresses one of the hottest issues in the field of AF ablation analyzing the rate of late PV reconnection after CB ablation, evaluating the most frequent PV sites of conduction recovery and then assessing procedural and biophysical indicators of PV reconnection in a large sample of patients having undergone the first repeat ablation following an index CB ablation procedure.

## 2 Methods

### 2.1 Aims

The aim of the study was to assess predictors of late PV reconnections after PV isolation initially achieved using CB ablation. Electric reconduction rate and gap localization were also taken into consideration as an end point. Sites of reconnections and procedural predictors of PV reconnections were also analyzed.

## 3 Study population

All patients having undergone a repeat catheter ablation at Heart Rhythm Management Centre (UZ Brussel, Brussels, Belgium) and Electrophysiology Unit of ZNA Heart Centre Middelheim (Antwerpen, Belgium) because of recurrent atrial tachyarrhythmia following PV isolation initially achieved with second-generation CB ablation for paroxysmal and persistent AF were consecutively included in this study. In this study population, all index procedures performed between 2015 and 2019 were included in the retrospective analysis. The study was approved by the Institutional Ethics Committee of our Institution. Data related to clinical history, imaging characteristics, interval history between index and repeat ablations, repeat ablation findings were collected.

## 4 Index procedure

Our standard preprocedural management and ablation have been previously described in detail [12], [14]. After a single transseptal puncture, left atrial (LA) access was gained, through a steerable 15 Fr sheath (FlexCath Advance®, Medtronic©) and an inner lumen mapping catheter (ILMC; Achieve, Medtronic©) was advanced and positioned in each PV ostium. A 28 mm CB-A (Arctic Front AdvanceTM, Medtronic©) was advanced inflated and positioned at PV ostium. Contrast injection was performed to check for optimal vessel occlusion. The ablation sequence was: left superior PV (LSPV) first, followed by the left inferior PV (LIPV), right inferior PV (RIPV), and right superior PV (RSPV). Cryoablation was started only once vessel occlusion was deemed satisfactory. Cryo-applications lasted at least 180 s. Pulmonary vein activity was recorded with the ILMC at a proximal site in the ostium before ablation in each vein. During ablation, if PV potentials were visible during energy delivery, time to isolation was recorded when PV potentials completely disappeared or were dissociated from left atrial activity. A single 3-minute freeze was applied for each vein and a second freeze was delivered in the case of failure to isolate the PV after the first cycle, in the case of nadir temperature greater than −35 °C, and in the occurrence of early PV reconnection. Durable PV isolation was assessed at least 20 min after ablation. Successful PV isolation was defined as an absence of all PV potentials or their dissociation from an atrial activity.

Additional cryo-applications were not carried out if the veins were already isolated. Entrance and exit block of the PV were performed to confirm electrical isolation using the ILMC. If needed, pacing from the distal and/or proximal coronary sinus was performed to distinguish eventual far-field atrial signals from PV potentials recorded on the mapping catheter, respectively, for left- and right-sided PVs. Moreover, after having retrieved the 15 Fr sheath to the right atrium while keeping the ILMC in the LSPV, a bipolar catheter was introduced through the transseptal access in the left atrial appendage (LAA), and pacing was performed in order to distinguish eventual far-field of LA electrical activity from PV potentials.

In order to avoid phrenic nerve palsy (PNP), a decapolar catheter was inserted in the right subclavian vein and diaphragmatic stimulation was achieved by pacing the ipsilateral phrenic nerve with a 1200-ms cycle and a 20-mA output. During the whole procedure, activated clotting time was maintained > 250 s by supplementing heparin infusion as required. Total procedure duration was considered as from having obtained femoral venous access to catheter removal.

## 5 Repeat ablation procedure

All repeat ablations were performed using radiofrequency energy. Femoral site access was obtained, and intravenous heparin was administered to maintain activated clotting times > 300 s.

After double transseptal puncture, 3D electroanatomical map (EAM) of left atrium was acquired with a circular mapping catheter (Carto, Biosense Webster© or NavX, St. Jude Medical©). PV isolation was checked and, if a PV was not isolated, radiofrequency applications were performed with an open irrigated tip catheter with contact‐force (CF) monitoring (Thermocool®, SmartTouch™, Biosense Webster©; Tacticath®, Endosense, St. Jude Medical©) in power‐controlled mode with a power limit of 35 W and maximum temperature of 48 °C. A 25 W power limit was used for posterior sites.

Late PV reconnection was defined as an LA–PV electric reconduction observed at the time of the repeat procedure. The location of gaps along previously deployed circumferential lesions was defined as the site of late PV reconnection in 4 different anatomic regions of the PV antrum: antero-superior, antero-inferior, posterior-inferior, and posterior-superior. The endpoint of PV isolation was defined as bidirectional block within a waiting time of 20  min after last application.

Additional substrate modification was performed in patients found to have durable PV isolation, non‐PV focal triggers initiating AF, or macro-reentrant atrial tachycardia or flutter, in accordance with consensus guidelines. Non PV-ablation involved the left atrial roof, floor and posterior wall, mitral isthmus, cava-tricuspid isthmus (CTI), superior vena cava, coronary sinus was performed at the discretion of the operator. Target contact force was between 5 and 20 g at all sites. Ablation duration was guided by the elimination of local electrograms and demonstrable entrance block following complete circumferential ablation. Acute procedural success was defined as electric isolation of PVs, confirmed by entrance block to individual PVs, and a line of bidirectional block when linear ablations were performed.

## 6 Follow up

After discharge, patients were scheduled for follow-up visits with baseline ECG and 24-hour Holter recordings at 1, 3, 6, and 12 months. Any symptoms after ablation were deemed as deserving a Holter monitoring. All reports of Holter or ECG recordings having been performed in referring centers were sent to our center for confirmation of the diagnosis of atrial tachyarrhythmia recurrence. Furthermore, telephone calls were performed during the follow-up. All documented episodes of atrial tachyarrhythmias lasting>30 s, with standard ECG or 24-hour ECG Holter monitoring and during both planned and symptom-driven consultation, were considered as a recurrence.

### 6.1 Statistical analysis

Categorical variables are expressed as absolute and relative frequencies, whereas continuous variables are expressed as mean ± SD. Comparisons of continuous variables were done with a Student *t* test and binomial variables with χ^2^ or Fisher test as appropriate. Factors predicting PV reconnections were identified by univariate and multivariable analyses using the logistic regression model.

A 2-tailed probability value of < 0.05 was deemed significant. Statistical analyses were conducted using the SPSS software (SPSS v22, Chicago, IL).

## 7 Results

A total of 300 consecutive patients (189 males, 63%; mean age 63.0 + 11.1 years) underwent a repeat ablation for recurrent symptomatic atrial tachyarrhythmias after an index PV isolation procedure by means of CB ablation (Table 1). The total amount of index CB procedures was 2158 and the follow up showed a total of 667 recurrences of atrial tachyarrhythmias (589 AF and 78 atrial flutters/tachycardias)”.

**Table 1:** Clinical characteristics of the study population.

	Total procedures (n = 300)
Male gender	189 (63%)
Age, years	63.0 ± 11.1
BMI, Kg/m^2^	27.5 ± 4.6
Hypertension	144 (48%)
Diabetes mellitus	26 (8.7%)
Previous cerebrovascular events (TIA, stroke)	35 (11.7%)
LVEF, %	56.2 ± 8.3
LA size, mm	44.2 ± 6.7
Paroxysmal AF	199 (66.1%)
Procedure duration, minutes	145.0 ± 50.7
Fluoroscopy duration, minutes	17.5 ± 10.3

Categorical variables are expressed as absolute and percentage (in brackets). Continuous variables are expressed as mean ± SD. BMI: body mass index. TIA: transient ischemic attack. LVEF: left ventricular ejection fraction. LA: left atrial. AF: atrial fibrillation.

The redo procedures were performed after 18.2 ± 10.8 months (median 15 months; range 1 – 68 months) from the index procedures. Table 1 shows baseline clinical characteristics of the study population according to the ablation strategy. The mean LA diameter for the total cohort was 44.2 ± 6.7 mm. A 4-PV pattern was present in 236/300 patients (78.7%), while a discrete left common ostium (LCO) could be observed in 43 subjects (14.3%) and a right middle PV was detected in 21 patients (7%). At presentation for repeat ablation, the majority were in normal sinus rhythm (n = 217, 72.3%) and taking anti-arrhythmic drugs (AADs; 85.3%). In detail, 164 patients were taking beta blockers (54.7%), 122 were taking IC class AADs (40.7%), 54 were on amiodarone (18%), 38 were on sotalol (12.7%).

**Fig. 1:**
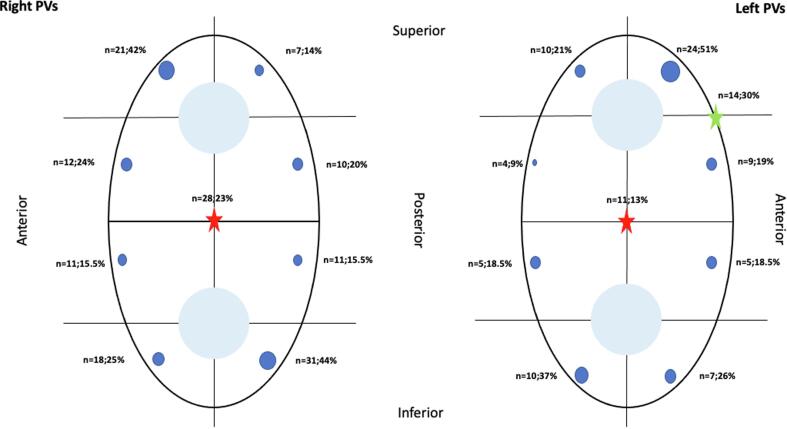
Distribution of reconducting gaps (blue dots) after second-generation CB ablation. The size of blue dots is related to the frequency of reconnection for that specific site. The red star indicates carina. The green star indicates the ridge between the ostium of LSPV and the left atrial appendage. PV: pulmonary vein. (For interpretation of the references to colour in this figure legend, the reader is referred to the web version of this article.)

Among all 1178 PVs, including 43 LCOs and 21 right middle PVs, 209 (17.7%) showed a late PV reconnection in 177 patients (1.18 per patient), at the time of repeat ablation procedure (Fig. 1). Overall, persistent PV isolation could be documented in 969 of 1178 PVs (82.3%). In 123 of 300 patients (41%), persistent isolation could be demonstrated in all PVs, whereas PV reconnection could be documented in 177 patients (59%). According to the PV location, different rates of persistent isolation could be documented: 210 LSPVs (81.7%), 228 LIPVs (88.7%), 250 RSPV (83.3%), 229 RIPVs (76.3%), 34 LCOs (79.1%), 18 right middle PVs (85.7%). Among all 209 reconnecting veins, 124 (59%) were in right-sided PVs, whereas 85 (41%) were in left-sided PVs. Only 29 of 257 (11%) reconnections were located in the LIPV, therefore, representing the less frequent site of conduction gap. Spatial distribution of late conduction gaps per PV region is shown in Fig. 1.

As the Fig. 1 shows, the LSPV mostly reconnected in the superior-anterior portion (51% of the overall gaps of this vein) while the LIPV exhibited the inferior and posterior portion as the most frequent side of reconnection (37%). Of note, the ridge between LA appendage and LSPV was reconnected in 14 cases (29.8% among the overall reconnections in LSPV) and the carina between the left PVs was found reconnected in 11 cases (12.8% of all reconnections in left PVs). Similarly, the RSPV showed a preferential area of reconnection in the superior-anterior portion (42% of all reconnections) while the RIPV had the inferior-posterior portion mostly reconnected (43.7% of all reconnections). The carina between the right PVs was found connected in 28 cases (23.1% of all reconnection in right PVs).

The LCOs were mostly reconnected in the inferior portion (6/9, 67%); 3 right middle PVs out of 21 (14.3%) still exhibited real time recordings but they were all small and diffusely reconnected without a specific, identifiable portion.

The occurrence of late PV reconnection was associated with warmer nadir temperature (-44.9 ± 18.2 vs −49.2 ± 8.5 °C, p = 0.003), longer time to isolation (42.1 ± 22.0 vs 35.2 ± 22.1 s, p < 0.001) and failure to achieve −40 °C within the first 60 s of application (88/209, 42% vs 271/969, 28%; p < 0.001) during the index procedure (Table 2). In addition, the group of PV reconnection exhibited longer freezing times to attain −40 °C (62.2 ± 31.1 vs 55.5 ± 23.5, p = 0.0005). However, no difference could be observed in mean rewarming time among veins showing or not PV reconnections (p = 0.4). No statistically significant difference could be also observed in the other procedural aspects such as mean number of applications and real-time recordings (respectively, p = 0.2 and p = 0.8).

**Table 2:** Index procedure characteristics in relation to PV isolation.

	PV isolated (n = 969)	PV reconnected (n = 209)	P value
Mean number of applications	1.20 ± 0.41	1.24 ± 0.43	0.2
Real time recordings, n (%)	649 (67%)	138 (66%)	0.8
Time to PV isolation, s *	35.2 ± 22.1	42.1 ± 22.0	<0.001
Time to −20 °C, sec	23.1 ± 4.3	23.3 ± 4.6	0.5
Time to −40 °C, sec	55.5 ± 23.5	62.2 ± 31.1	0.0005
−40 °C in 60 s, n (%)	698 (72%)	121 (58%)	0.04
Nadir temperature, °C	−49.2 ± 8.5	−44.9 ± 18.2	0.0001
Total rewarming time, s	43.1 ± 18.5	42.0 ± 19.7	0.4

Data are expressed in mean ± SD or absolute number (percentage). PV: pulmonary vein. *data available for only 787 pulmonary veins.

In the univariate analysis, time to PV isolation, −40 °C achievement within 60 s, time to −40 °C and nadir temperature were significantly associated with LA–PV reconduction (Table 3). Multivariable analysis confirmed time to PV isolation, nadir temperature and failure to achieve − 40 °C within 60 s as independent predictors of late PV reconnections (Table 3).

**Table 3:** Univariate and multivariable analysis indicating factors predicting late PV reconnection.

	OR (95% CI)	P value
**Univariate analysis**		
Time to PV isolation	1.04 (1.02–1.07)	<0.001
Nadir temperature	1.03 (1.01–1.05)	0.02
Time to −40 °C	1.01 (0.99–1.02)	0.055
−40 °C in 60 s	1.76 (1.02–3.00)	0.04
**Multivariate analysis**		
Nadir temperature	1.02 (1.01–1.07)	0.03
−40 °C in 60 s	1.86 (1.02–5.60)	0.05
Time to PV isolation	1.03 (1.01–1.05)	0.01

CI: confidence intervals. PV: pulmonary vein. The odds ratio for time to PV isolation considers every second increase. The odds ratio for nadir temperature considers every centigrade decrease.

All redo procedures were performed with an RF irrigated-tip catheter guided by contact-force monitoring using a 3D electro-anatomical mapping system. The repeat procedure was performed because of AF recurrence in 257 of 300 patients (85.7%), whereas a regular atrial tachycardia was documented in 43 patients (14.3%). Atrial tachycardias presented as roof-dependent left flutter in 15 patients (34.9%), mitral-isthmus–dependent flutter in 11 patients (25.6%), PV-dependent flutter/tachycardia in 13 patients (30.2%; 8 from right PVs and 5 from left PVs) and 4 (9.3%) showed a focal trigger from right atrium (n = 1) and superior vena cava (n = 3). Re-PV isolation and substrate ablations were successfully performed in all patients. Cryoablation lesions were located at the PV antrum and point-by-point RF applications focused at the gaps without the need to ablate the whole quadrant. Among 127 patients (42.3%) receiving additional RF applications, 40 (31.5%) were treated with a cava-tricuspid line, 71 (55.9%) with a roof line, 49 (38.6%) with a mitral isthmus line, 10 (7.9%) with superior vena cava isolation and 56 (18.7%) received a posterior box isolation. None of the patients died or experienced cerebrovascular events in the periprocedural period. After a mean follow-up of 12.7 ± 8.3 months, 44 (14.7%) continued to experience symptomatic AF episodes. None of those presenting with atrial tachycardia experienced further arrhythmic recurrences.

In 18 of 44 (41%) patients, a third ablation procedure was performed. In 14 of 18 (78%) patients, all PVs were still isolated. Among patients presenting reconnections, 5 sites of reconnections were localized in the LSPV (n = 2, 40%), LIPV (n = 1, 20%), RIPV (n = 2, 40%). Thus, a total of 67 of 72 (93%) patients identified that PVs were still isolated after the second procedure.

## 8 Discussion

The main findings of the present study are that (1) PV reconnection rate assessed during repeat ablations is 18% (1.18 PVs/patient); (2) the most frequent sites of reconnections are the superior-anterior portions for the upper PVs and the inferior-posterior portions for the lower PVs; and (3) time to PV isolation, nadir temperature and failure to achieve − 40 °C within 60 s are independent predictors of PV reconnections.

Our results are consistent with the majority of previous findings following CB ablation. In 18 patients admitted for re-ablation because of recurrent atrial tachyarrhythmias, Bordignon et al[10] found persistent electrical isolation in 55 of 71 (77%) previously isolated PVs; similarly, Heeger et al [11] and Ciconte et al [12] found comparable rate of persistent electrical PV isolation (respectively, 69% and 78%) in larger samples of patients (respectively 66 patients and 258 PVs; 29 patients and 115 PVs). A persistent isolation of all PVs was found in a range between 26% and 33% of patients [10], [11], [12]. These abovementioned studies showed a low rate of late PV reconnection following second-generation CB ablation ranging from 0.89 to 1.25 PVs per patient. A more recent study [13] combining data from two high-volume centers (Mercy General Hospital, Sacramento, USA and Vrije Universiteit Brussel, Brussels, Belgium) retrospectively analyzed 112 patients who underwent a repeat procedure 14 ± 3 months after CB ablation procedure; among 435 PVs, the authors found durable PV isolation in 324 of them (74.5%) and a mean number of PV reconnections of 0.99 per patient.

The present study have analyzed 300 patients having undergone re-do ablation because of recurrence of atrial tachyarrhythmias following an index procedure of CB ablation. We confirmed the evidence of a very low rate of PV reconduction (1.19 PV reconnection per patient); a persistent isolation of all PVs was found in 41% of our patients. A durable PV isolation was evidenced in in 969 of 1178 analyzed PVs (82%).

However, some authors found higher rates of PV reconduction following CB ablation [15], [16], [17]. Daimee et al [16] analyzed 300 patients having undergone their first repeat ablation for symptomatic, recurrent AF; among 45 patients of this cohort having undergone CB ablation as index procedure, they found a mean number of PV reconnection per patients of 2.9 ± 1.2. Similarly, Kettering et al [15] analyzed 30 patients having undergone a redo procedure following cryoablation and they found a mean number of 2.9 reconnected PVs per patient. Nolasco et al [17] evaluated 27 re-ablation following CB ablation as index procedure and they found 1.6 ± 0.4 reconnected PVs per patient.

Generally, our findings showed that preferential sites of PV reconduction following CB ablation were located in the superior and anterior portions of the upper PVs and in the inferior-portions of the lower PVs. Of note, the reconnection was found at the ridge between LSPV and left atrial appendage in one third of all reconnections of the LSPV. In addition, carina between upper and lower septal veins was found to be reconnected in one fourth of the whole reconnections of right PVs.

We might hypothesize that sites of frequent reconnections represent areas where CB does not reach enough contact resulting in a shallow lesion. This might occurs because PV antrum shape is not perfectly circular in many cases, and size of each PV is always variable, therefore the pressure of the CB is not homogeneous over the entire endocardial surface [17]. Moreover, some areas of the PV antrum with a thicker wall, such as the endocardial ridge, are prone to have non-transmural lesions; in these areas, an adequate cryo-lesion should probably attain a sufficient depth to provide a durable isolation [17].

As already largely proven, a late PV reconnection is more frequent in those veins with warmer nadir temperatures and a delayed time to isolation.

In detail, longer time to isolation and failure to achieve − 40 °C within 60 s seem to be independent predictors of PV reconnections. Time to isolation has been widely proven as the strongest predictor of durable PV isolation and is directly related to the PV occlusion [12], [13], [14]. A colder nadir temperature has been also shown to be associated with a durable PV isolation [18].

The freezing drop within 60 s is crucial for several reasons [12]. The lesion formation induce by CB ablation is based on the generation of hypothermia at the catheter–tissue interface. Progressive cooling below −40 °C results in the formation of intracellular ice crystals, which is the first step in ensuring adherence of the catheter to the tissue during the cryo-lesion. Moreover, full-flow cryo-refrigerant is usually achieved within 1 min, and at that time the slope of the curve starts to plateau. Therefore, during hypothermia the catheter adheres to the tissue achieving greater stability, thus, eliminating the brushing effect that occurs during beat-to-beat heart motions and with respiratory variations [12].

However, all these parameters depend on PV occlusion; the highest is the degree of PV occlusion the steeper is the freezing drop resulting in faster time of isolation and colder temperature nadir. As widely demonstrated, the PV occlusion is the main feature to be pursued during the cryoablation.

To the best of our knowledge, our series is the largest sample of redo procedures investigating PV reconnection following CB ablation. The present findings actually confirm the low incidence of reconduction gaps and similar preferential sites of PV reconnection detected by preliminary studies using second-generation CB technology.

## 9 Limitations

The present study is a retrospective and non-randomized analysis. A further limitation of this study consists in the fact that only patients with documented recurrence of atrial tachyarrhythmias after the index procedures have been included. Therefore, the rate of late PV recovery in our patient cohort might represent an overestimation of the real incidence of late PV reconnections in individuals having undergone CB ablation. Another important limitation is that redo procedures were performed with circular mapping catheters; a high-density mapping with novel mapping catheters or other mapping systems (such as Boston Rhythmia) might have potentially found more gaps of PV reconnection.

## 10 Conclusions

The rate of late PV reconnection assessed during repeat ablation is low (1.18 PVs/patient). The most frequent sites of reconnections are the superior-anterior portions for the upper PVs and the inferior-posterior portions for the lower PVs. Faster time to PV isolation, colder nadir temperatures and achievement of − 40 °C within 60 s are associated with durable PV isolation.

## Declaration of Competing Interest

GBC reports speaker fees for Medtronic, Biotronik, Biosense Webster, and Abbott; teaching honoraria from Medtronic and Biotronik; proctoring honoraria from Medtronic; C.d.A. reports speaker fees for Medtronic, Biotronik, Biosense Webster, Abbott, and Boston Scientific; teaching honoraria from Medtronic, Biotronik, Abbott, and Boston Scientific; proctoring honoraria from Medtronic, Abbott, and Biotronik. Funding: research support from the Heart Rhythm Research Center.
